# Aseptic Raman spectroscopy can detect changes associated with the culture of human dental pulp stromal cells in osteoinductive culture

**DOI:** 10.1039/c5an01595b

**Published:** 2015-09-16

**Authors:** Adam Mitchell, Lorna Ashton, Xuebin B. Yang, Royston Goodacre, Matthew J. Tomlinson, Alistair Smith, Jennifer Kirkham

**Affiliations:** a University of Leeds , Department of Oral Biology , Leeds School of Dentistry , Leeds , UK . Email: J.Kirkham@leeds.ac.uk; b Department of Chemistry , Faraday Building , Lancaster University , Lancaster , UK; c School of Chemistry and Manchester Institute of Biotechnology , University of Manchester , Manchester , UK; d Avacta Group plc , Thorpe Arch Estate , Wetherby , UK

## Abstract

There is an unmet need for the non-invasive characterisation of stem cells to facilitate the translation of cell-based therapies. Raman spectroscopy has proven utility in stem cell characterisation but as yet no method has been reported capable of taking repeated Raman measurements of living cells aseptically over time. The aim of this study was to determine if Raman spectroscopy could be used to monitor changes in a well characterised cell population (human dental pulp stromal cells (DPSCs)) by taking repeated Raman measurements from the same cell populations in osteoinductive culture over time and under aseptic conditions. DPSCs were isolated from extracted premolar teeth from 3 consenting donors. Following *in vitro* expansion, DPSCs were maintained for 28 days in osteo-inductive medium. Raman spectra were acquired from the cells at days 0, 3, 7, 10, 14 and 28. Principal component analysis (PCA) was carried out to assess if there was any temporal spectral variation. At day 28, osteoinduction was confirmed using alizarin red staining and qRT-PCR for alkaline phosphatase and osteocalcin. Alizarin red staining was positive in all samples at day 28 and significant increases in alkaline phosphatase (*p* < 0.001) and osteocalcin (*p* < 0.05) gene expression were also observed compared with day 0. PCA of the Raman data demonstrated trends in PC1 from days 0–10, influenced by protein associated features and PC2 from days 10–28, influenced by DNA/RNA associated features. We conclude that spectroscopy can be used to monitor changes in Raman signature with time associated with the osteoinduction of DPSCs using repeated measurements *via* an aseptic methodology.

## Abstract 2

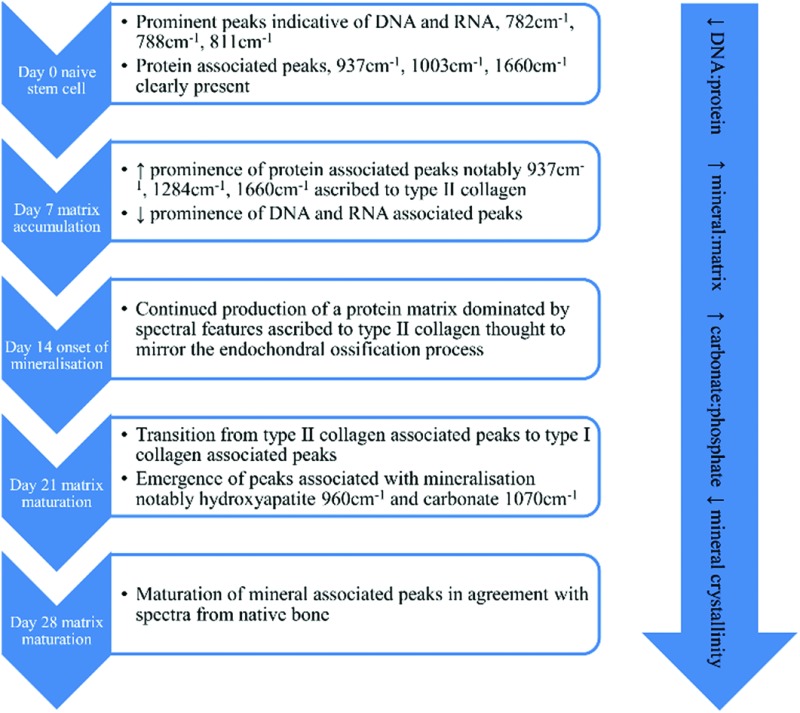
There is an unmet need for the non-invasive characterisation of stem cells to facilitate the translation of cell-based therapies.

## Introduction

The field of tissue engineering and regenerative medicine has advanced rapidly since its inception by Langer and Vacanti in 1993,^1^ with clinical trials for the treatment of numerous conditions underway.^2^ Stem cells are an important component of the tissue engineering toolkit, from pluripotent embryonic stem cells and induced pluripotent stem cells to multipotent somatic stem cells including mesenchymal stem cells (MSCs) and haematopoietic stem cells (HSCs). The use of MSCs avoids the ethical concerns associated with embryonic stem cell research and whilst MSCs cannot differentiate along as many cell lineages they still possess demonstrable capacity for differentiation into osteoblasts, chondrocytes, adipocytes,^3^ tenocytes,^4^ hepatocytes^5^ and neural cells.^6^ Such directed differentiation of MSCs and validation of their resulting phenotype requires significant expansion of stem cell cultures and testing with invasive and/or destructive methods that preclude their subsequent use in clinical applications. Non-invasive methods that can reliably monitor stem cell differentiation could reduce the need for *in vitro* expansion and save researchers a great deal of time and resources when conducting their experiments. Raman spectroscopy is one such potential methodology for *in situ* analysis.

**Fig. 1:**
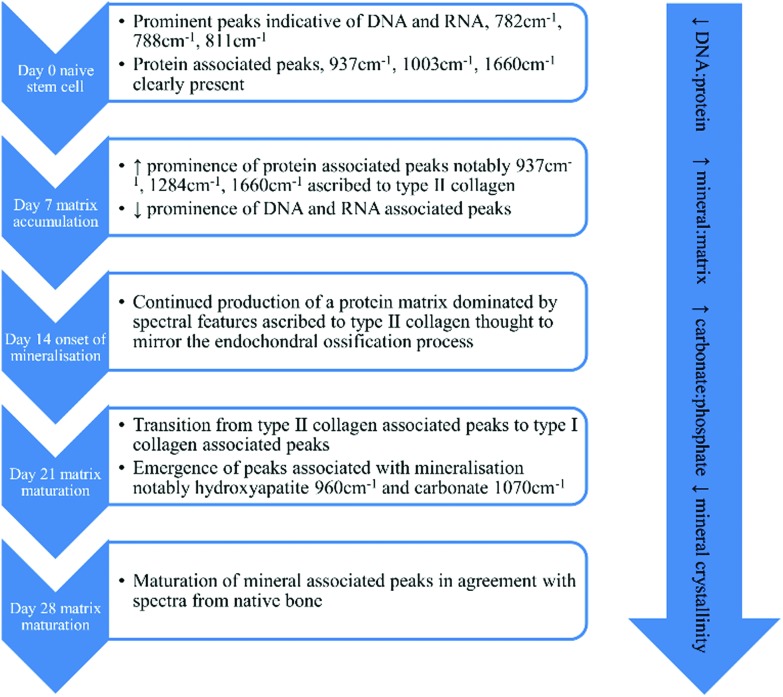
Proposed timeline of events outlining the osteogenic differentiation process of stem cells using Raman spectroscopy, based on data from ref. 9 and 11–15.

Raman spectroscopy has the capacity to be both non-invasive and non-destructive and utilises a monochromatic light source to determine sample chemistry. Upon interaction with the sample a small fraction of the light, approximately 1 in 10^6^ to 10^8^ photons,^7^ is shifted in wavelength with respect to the incident laser. Many chemical bonds in the sample cause unique Raman shifts such that the resultant spectrum can be considered to be a ‘molecular fingerprint’ that is unique to the sample under analysis. Several recent publications have described the use of Raman spectroscopy to determine cell viability,^8–10^ to identify general markers of cell differentiation,^9,11^ to track differentiation to an osteoblastic phenotype^12^ and to elucidate changes in extracellular matrix calcification and/or mineralisation concomitant with osteoblastic differentiation.^13–16^ These studies have identified a signature profile for the differentiation of stem cells down the osteogenic lineage based on the emergence and relative ratios of peaks in their Raman spectra illustrated in Fig. 1. Whilst these data have highlighted the potential usefulness of Raman spectroscopy in stem cell phenotyping, to date repeated measurements of the same cell population in long term culture have been precluded due to the need to maintain sterility, yet the ability to deliver this would be greatly advantageous when developing cell-based regenerative therapies. Raman scattering efficiency is very poor and the distance between the sample and the microscope objective needs to be as small as possible for maximum sensitivity. In order to maximise the potential of Raman spectroscopy as a tool for non-invasive stem cell characterisation over time, studies need to be conducted under aseptic conditions while maintaining the strength of the Raman signal. This would then ultimately permit early/predictive identification of differentiation such that stem cells may be used in further downstream applications.

In this study, our aim was to develop a novel methodology that permits the repeated acquisition of Raman spectra from the same cell cultures without prejudicing culture sterility, including investigating whether or not the Raman acquisition process might adversely affect the cells’ behaviour. Our hypothesis was that Raman might usefully indicate early phenotypic shifts in a mixed stromal cell population during osteoinduction. We elected to use dental pulp stromal cells (DPSCs) for this work as their differentiation behaviour is well characterised and documented within the literature. DPSCs are one of several cell sources that are more broadly classed as MSCs and were first isolated by Gronthos *et al.* (2000).^17^ They have since been shown to differentiate down multiple lineages including osteogenic, dentinogenic, adipogenic, chondrogenic, myogenic and neurogenic and express many of the cell surface markers used to characterise bone marrow derived MSCs.^18–22^ However, it should be noted that the propensity for differentiation along a specific lineage can vary amongst MSCs derived from different sources.^21,23^ DPSCs were chosen specifically for this study as they have a proven predisposition toward the osteogenic/dentinogenic lineage,^24^ presenting a reliable and well documented sample for evaluating our new method. We envisage that Raman spectroscopy would be used as part of a minimally invasive approach to tissue engineering and as such we used unsorted stromal cells from the dental pulp as opposed to a fluorescence activated cell-sorted (FACS) enriched stem cell population. We osteoinduced DPSCs and acquired Raman spectra at regular time intervals throughout osteoinduction culture period to determine both technical feasibility and identify whether changes in the Raman spectra might be prejudicial to the well documented differentiation of DPSCs under these conditions.

## Materials and methods

### Raman spectroscopy of dental pulp stromal cells (DPSCs) in osteogenic culture

#### DPSC isolation and culture

Human molars were obtained from one male and two female donors, aged 9, 22 and 35 year, by informed written consent (in the case of minors, consent was obtained through the parent/guardian) through the Leeds School of Dentistry Research Tissue Bank as authorised by the Dental Research Ethics Committee (DREC 07/H1306/93+5). DPSCs were isolated by the collagenase/dispase digestion method previously described by Gronthos *et al.* ^17^ and maintained in basal medium, α-MEM containing 10% foetal calf serum (FCS) (Biosera, Ringmer, UK), 1% 10 000 units per 10 mg mL^–1^ penicillin/streptomycin solution (Sigma-Aldrich, Gillingham, UK) and 1% 200 mM l-glutamine (Sigma-Aldrich). Cultures were incubated at 37 °C, 5% CO_2_ and routinely passaged. DPSCs at passage 3 were used in all subsequent experiments.

#### DPSC phenotyping

DPSCs were treated with 0.25% trypsin/0.02% EDTA (Sigma-Aldrich) and centrifuged to generate a cell pellet. Cells were labelled with 10 μL FcR blocking solution (Miltenyi Biotec, Bergisch Gladbach, Germany) and various antibodies (see below, 10 μL per 1 × 10^6^ cells unless stated) in a total volume of 100 μL labelling buffer (PBS, 2 mM EDTA (Alfa Aesar, Heysham, UK) and 0.5% (w/v) BSA (Sigma-Aldrich)) for 20 minutes at room temperature in the dark. Following labelling, 900 μL labelling buffer was added to each sample before centrifugation and resuspension in 500 μL labelling buffer. Samples were then analysed using an LSRII flow cytometer (BD Biosciences, San Jose, CA) running FACSDiva 8.0 software (BD Biosciences), subsequent data analysis was performed using Kaluza 1.3 (Beckman Coulter, Inc., Miami, FL). Antibodies used were: CD29-Alexa Fluor 488 (5 μL per 1 × 10^6^ cells), CD34-FITC (5 μL per 1 × 10^6^ cells), CD44-FITC, CD73-PE (2 μL per 1 × 10^6^ cells), CD90-APC and CD166-PE (all Biolegend, San Diego, CA). All threshold values were obtained by analysing autofluorescence of non-labelled hDPSCs and reactivity of isotype matched controls. Events were gated based on forward and side scatter and colour compensation was performed.

#### Quartz customised flask preparation

**Fig. 2:**
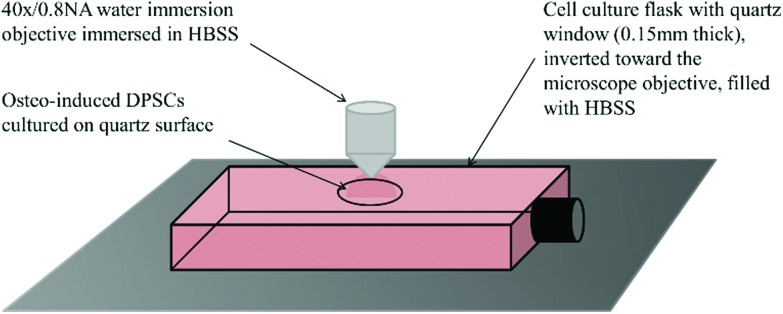
Schematic of a quartz window customised flask prepared for Raman spectroscopy. The microscope used to acquire Raman spectra uses an upright turret, therefore flasks had to be inverted toward the microscope objective. This necessitated the filling of the flask with HBSS to prevent the cells from desiccating during analysis.

Customised cell culture flasks with quartz windows (Fig. 2), suitable for Raman spectroscopy, were prepared by drilling 1 cm diameter holes in 25 cm^2^ cell culture flasks. Flasks were washed several times with sterile pure water to remove debris and finally sterilised with 70% ethanol. Heat sterilised quartz discs (Global Optics, Bournemouth, UK), 14 mm diameter × 0.15 mm thickness, were glued to the outside of the flasks using a UV cured methacrylate glue (Loctite, Hempstead, UK) and cured for 5 min under UV light. To ensure complete sterilisation, flasks were placed under a UV lamp for a further 90 min and periodically rotated to ensure penetration to all surfaces.

#### DPSC sample preparation and Raman spectroscopy

Sterile poly-l-lysine (0.1 mg mL^–1^, molecular weight, 70 000–150 000 (Sigma-Aldrich)) was used to coat the quartz surface of the customised flasks to enhance cell adhesion, as poly-l-lysine coatings have little/no effect on Raman spectra.^25^ The solution was then aspirated and the flask dried in an incubator at 37 °C overnight. DPSCs suspended in basal medium were pipetted directly on to the quartz surface at 1 × 10^5^ cells per cm^2^ (*n* = 3) and incubated overnight at 37 °C to adhere. The following day flasks were topped up with osteoinductive medium, (basal medium supplemented with 0.25 mM ascorbic acid, 10 mM β-glycerophosphate and 100 nM dexamethasone (all Sigma-Aldrich)). This was designated day 0. Each of the donor samples were cultured and analysed in triplicate. An equal number of samples were maintained in osteogenic conditions but did not undergo Raman analysis. These were used as controls for the sampling process. At no point during the cell culture period was there any evidence of bacterial or fungal contamination.

Prior to Raman analysis the cell culture medium was aspirated and flasks filled with Hanks balanced salt solution (HBSS) with glucose added to a final level of 0.45%. Flasks were placed on the stage of a Leica DMLM microscope attached to a Renishaw RM series Raman spectrometer and inverted toward the objective. A 40×/0.8 NA water immersion objective was immersed in a small drop of HBSS placed on the quartz surface. DPSCs were first brought into focus under transmitted white light then over-focused by 5 μm and switched to 785 nm light for spectrum acquisition. Spectra were acquired at days 0, 3, 7, 10, 14 and 28. At each time point 15 spectra were acquired over the wavenumber range 600–1800 cm^–1^ with an exposure time of 120 s and the power on the sample from the 785 nm laser was 66 mW. With 9 samples (three donors in triplicate) this produced a total of 135 spectra at each time point. All spectra were acquired from the DPSC monolayer even at later time points, despite the emergence of mineralised nodules, for consistency. Cell free sections of quartz were used to acquire the background spectrum. Following analysis the HBSS was aspirated and replaced with osteoinductive medium and the flasks returned to the incubator.

#### Data analysis

Pre-processing of spectra was performed in Grams/32 (Thermo Scientific, Waltham, USA) with further processing and validation by principal component analysis (PCA) performed in Matlab version R2013b. In Grams each individual spectrum was background subtracted using cell free areas of HBSS filled quartz customised flasks, *y*-axis normalised to the peak at 1003 cm^–1^ to correct for any minor unavoidable instrumental drift due to sampling over a long period of time, and smoothed using a 7 point Savitzky-Golay filter. All spectra were then exported to Microsoft Excel for further processing in Matlab. PCA was performed on all spectra from each individual donor and used to remove outlying spectra from each time point. The data were then averaged using one spectrum from each donor per time point. Finally second derivative spectra were calculated and further smoothing performed. PCA was then carried out to visualise the fully processed data. Such pre-processing was required to overcome intrinsic experimental constraints such as the longer working distance and the need to acquire spectra through an interface presented by working aseptically with quartz windowed flasks.

### Osteogenic and cell stress marker gene expression of DPSCs ± analysis by Raman spectroscopy determined by qRT-PCR

To confirm osteoinduction and determine any effects of Raman spectroscopy on the osteoinductive process, qRT-PCR was used to determine marker gene expression, RNA isolation and reverse transcription was performed on DPSCs following osteoinductive culture ± Raman spectroscopy analysis. Briefly, DPSCs were lysed by incubation with Trizol® (Invitrogen, Paisley, UK) for 20 min at room temperature. RNA was extracted from cell lysates using an RNeasy mini kit with additional DNase digestion (Qiagen, Crawley, UK) following the manufacturers recommendations. RNA concentrations were quantified using a NanoDrop spectrophotometer (Thermo Scientific). A High Capacity RNA-to-cDNA Kit (Applied Biosystems, Carlsbad, USA) was used to prepare 200 ng of cDNA in 20 μL reaction volumes as per the manufacturer's instructions. Reactions were run on a MJ Research PTC-100 Thermo Cycler, at 37 °C for 1 h and 95 °C for 5 min. qRT-PCR was performed using TaqMan gene expression assays (Applied Biosystems) for alkaline phosphatase (*ALP*) and osteocalcin (*OC*): assay numbers Hs01029144-m1 and Hs00609452-g1 respectively, to determine early and late osteogenic marker expression and for heat shock transcription factor 1 (*HSF1*), hypoxia inducible factor 1 (*HIF1A*) and lactate dehydrogenase (*LDH*): assay numbers Hs00232134-m1, Hs00153153-m1 and Hs00929956-m1 respectively, to investigate cell stress marker expression. Reactions were carried out in 96 well plates on a Roche LightCycler® 480 system. Optimal cDNA concentration was determined to be 5 ng/reaction by plotting standard curves with known cDNA concentrations. Data were normalised to *YWHAZ* assay number Hs00237047-m1, and analysed using the comparative cycle threshold method (ΔCT). *YWHAZ* was selected as it has been shown to be a far more reliable housekeeping gene compared with some of the more traditional housekeeping genes such as *GAPDH* and *ACTB.* ^26^ Three patient samples, one in duplicate, were analysed using both DPSCs that had undergone analysis by Raman spectroscopy and DPSCs cultured in parallel under the same conditions but without subjection to Raman spectroscopy after 28 days of osteoinductive culture. Samples were analysed in triplicate for a total *n* = 12 for each group. Samples from day 0 were used to provide a baseline. Graphs of the mean ΔCT ± SE were plotted in Microsoft Excel and statistical significance was determined by one-way ANOVA using GraphPad Instat 3 software.

### Extracellular matrix calcification of DPSC cultures ± analysis by Raman spectroscopy using alizarin red staining

Alizarin red staining was performed on day 28 DPSC cultures that had been analysed by Raman spectroscopy and those that had not (*n* = 2). In both cases, DPSCs had been cultured in osteoinductive medium as described above. Cell monolayers were washed twice with PBS and fixed in 10% neutral buffered formalin for 1 h at room temperature. Cell monolayers were incubated with 1% Alizarin red solution (Sigma-Aldrich) (pH 4.2), for 20 min at room temperature. The Alizarin red solution was then removed and excess stain washed away with repeated washes with de-ionised water. Cultures were then imaged using an Olympus BX50 microscope with accompanying Nikon DS-Fi1 camera.

## Results and discussion

### Phenotyping of undifferentiated DPSCs using flow cytometry

**Fig. 3:**
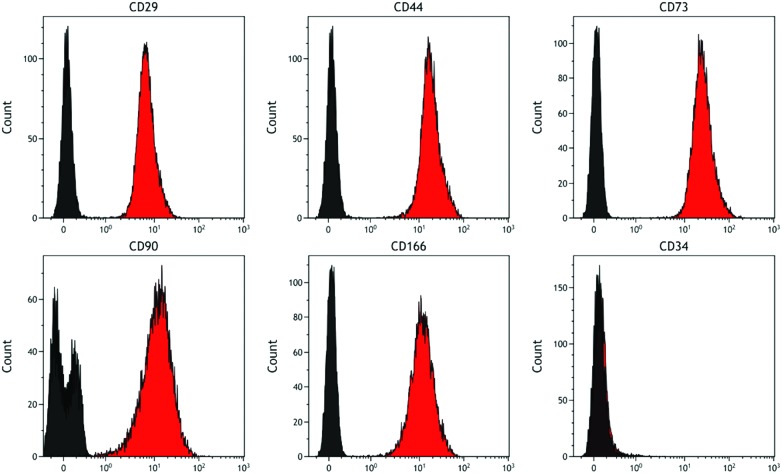
Representative histograms showing cell surface marker expression by DPSCs. Markers examined were CD29, CD44, CD73, CD90, CD166 and CD34. Data confirm the stromal phenotype of these cells as they were negative for the haematopoietic marker CD34 and positive for all the other markers tested (red histogram cells, black histogram isotype controls).

Analysis of DPSCs confirmed these cells possessed a stromal phenotype, being positive for the standard DPSC markers tested (including CD29, CD44, CD73, CD90, CD166) and negative for the haematopoietic marker CD34 (Fig. 3).

### Raman spectroscopy of DPSCs osteogenic culture

**Fig. 4:**
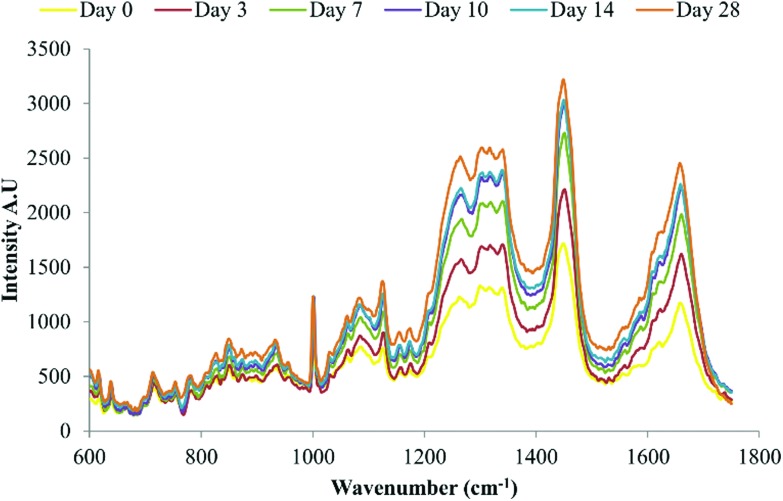
Average background subtracted spectra of osteo-induced DPSCs at days 0, 3, 7, 10, 14 and 28, *n* = 135 spectra per time point. Data from three separate donors examined in triplicate.

Raman spectroscopy has previously been used to demonstrate late stage osteogenic differentiation of stem cells by analysing putative mineralised nodules in the extracellular matrix.^13,15^ In this study DPSCs were repeatedly analysed by Raman spectroscopy with a focus on earlier time points *i.e.* prior to the emergence of mineralised nodules. Fig. 4 depicts the average spectra for each time point (day 0 to day 28) following subtraction of the signal from the (quartz) background. Many peaks typical of biological samples were clearly evident such as phenylalanine at 1003 cm^–1^, CH_3_, CH_2_ found in collagen at 1453 cm^–1^ and amide I at 1660 cm^–1^.

**Fig. 5:**
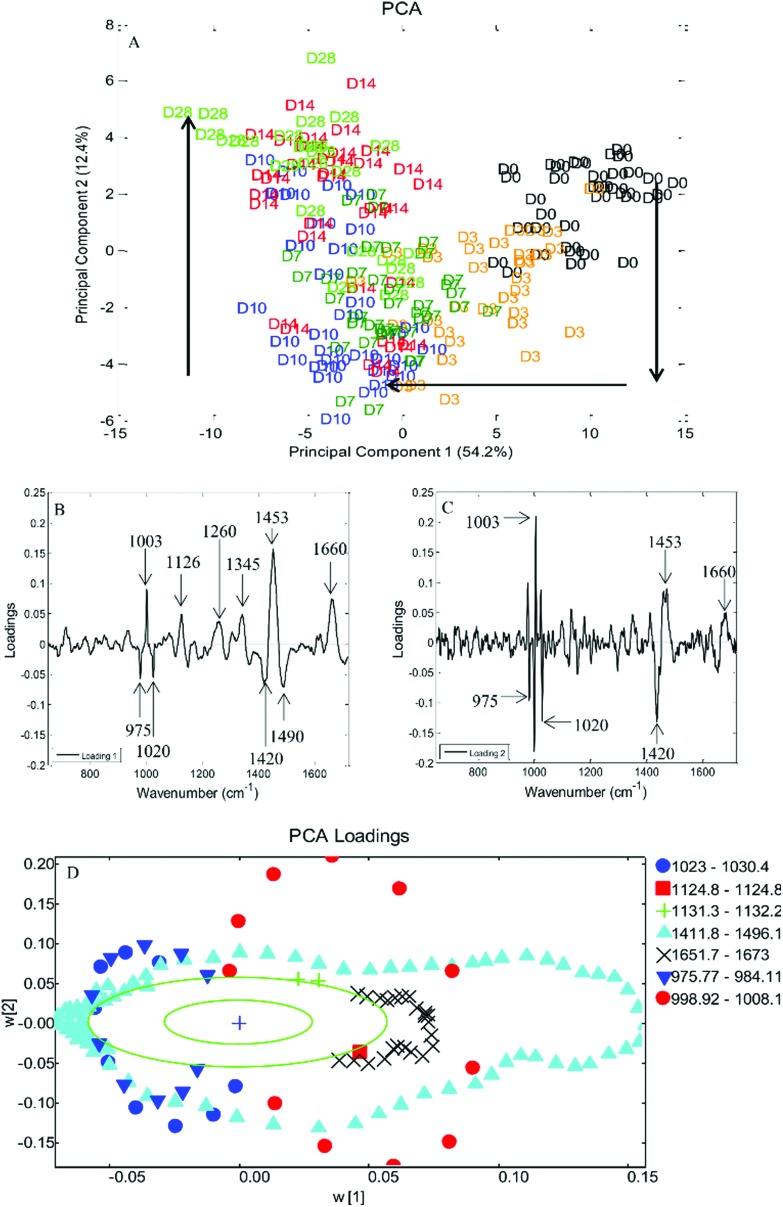
(A) Principal component analysis of the osteogenic differentiation of DPSCs at days 0, 3, 7, 10, 14 and 28. 2D scatter plot for principal components 1 and 2. Each position displayed is the average of three spectra, one from each donor, for every time point. There is a clear trend in PC1 between days 0 and 10 and a further trend in PC2 between days 0 and 10 and days 10 and 28, marked with arrows. (B + C) PCA loadings plot for PC1 and PC2 respectively, from the PCA of DPSCs that had been osteo-induced for 0, 3, 7, 10, 14 and 28 days. Significant positive and negative peaks are marked. (D) PCA loadings plot for PC1 *versus* PC2, the outer green circle indicates 95% confidence. In the legend are groups of wavenumbers from peaks that vary significantly.

Principal component analysis (PCA) was performed on spectra obtained from DPSCs from three donor samples, analysed in triplicate, which had been averaged, *n* = 135 spectra at each time point. PCA is a method of reducing the dimensionality of multivariate data into groups of potentially correlated variables called principal components (PCs). PC1 represents the group of variables responsible for the greatest amount of variation in the data and PC2 the group of variables responsible for the second greatest variation in the data and so on. In the case of Raman spectra such as those presented here the variables are the wavenumbers which in turn correspond to particular molecular species within the cells. Fig. 5A is a 2D scatter plot of PC1 and PC2 scores accounting for 54.2% and 12.4% of the variation in the data set respectively, this indicates that the wavenumbers contained within PC1 and concomitantly the molecular species they are associated with accounted for 54.2% of the variation in the data set and so on. The PCA scores plot demonstrated a clustering of spectra at each time point, illustrating that the spectral collection was reproducible. There was overlapping of spectra between adjacent time points as might be expected if a continuous phenotypic shift was occurring due to cell differentiation. Despite the overlapping of time points a clear trend was observed from day 0 to day 10 along PC1 while a trend from day 7 to day 28 was observed along PC2. Loadings plots can be drawn for each PC to illustrate those spectral features that are responsible for the variance in that PC. Peaks on the same side of a loading plot will be behaving similarly, whilst peaks on opposite sides of a loading plot will be behaving differently with respect to their scores within a given PC. Fig. 5B + C illustrate the loading plots for PC1 and PC2. The loading plot for PC1 showed positive peaks for wavenumbers corresponding to amide bonds (1260 cm^–1^ and 1660 cm^–1^), CH_3_, CH_2_ from collagen (1345 cm^–1^ and 1453 cm^–1^), phenylalanine (1003 cm^–1^) and lipids (1126 cm^–1^) whilst wavenumbers corresponding to ribose (975 cm^–1^ and 1020 cm^–1^), the nucleic acids guanine and adenine (1420 cm^–1^) and DNA (1490 cm^–1^) had negative peaks. Similarly the loading plot for PC2 had positive peaks at 1003 cm^–1^, 1453 cm^–1^ and 1660 cm^1^ with negative peaks at 975 cm^–1^, 1020 cm^–1^ and 1490 cm^–1^. Putative peak assignments were based on Movasaghi *et al.* ^27^ As the above individual PCs both use the same areas it may be hard to differentiate which are the important vibrations with respect to time, therefore PC1 was plotted against PC2 (Fig. 5D). Each data point represents the loadings value of PCs 1 and 2 for a given wavenumber. When plotted as such the position of a given wavenumber on the plot when transposed on the original scores plot indicates where that wavenumber varies most greatly. This loadings plot showed that two protein associated peaks at 1453 cm^–1^ and 1660 cm^–1^ transpose along the trend in PC1 from days 0 to 10, whilst two DNA/RNA associated peaks 975 cm^–1^ and 1020 cm^–1^ transpose along the trend in PC2 from days 10 to 28 as did the phenylalanine peak at 1003 cm^–1^.

**Fig. 6:**
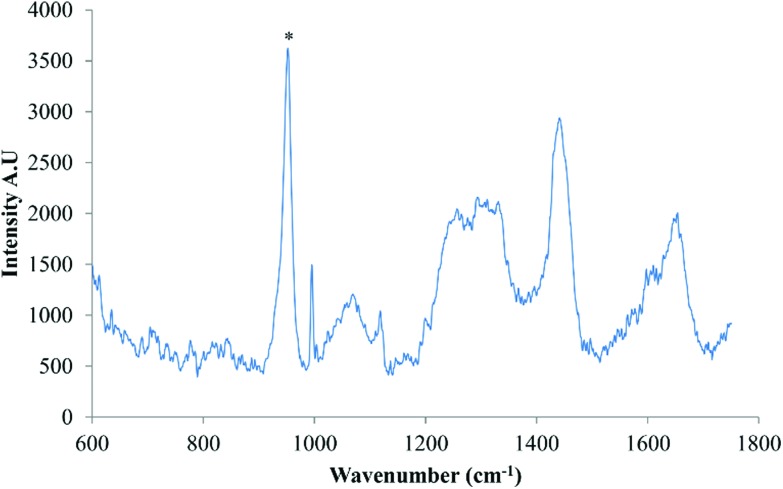
Average background subtracted spectra of osteo-induced DPSCs at day 28 acquired from putative mineralised nodules, *n* = 5. The well characterised mineral associated region at ∼955 cm^–1^–964 cm^–1^ is clearly evident, marked *.

Finally, to confirm whether osteoinduction of DPSCs was evident by day 28, several Raman spectra were acquired from putative mineralised nodules, (Fig. 6). The well characterised mineral associated region, 955 cm^–1^–964 cm^–1^, described in several previous studies,^13–15^ was clearly evident.

### Effect of Raman spectroscopy on DPSC osteogenic marker gene expression and extracellular matrix deposition

**Fig. 7:**
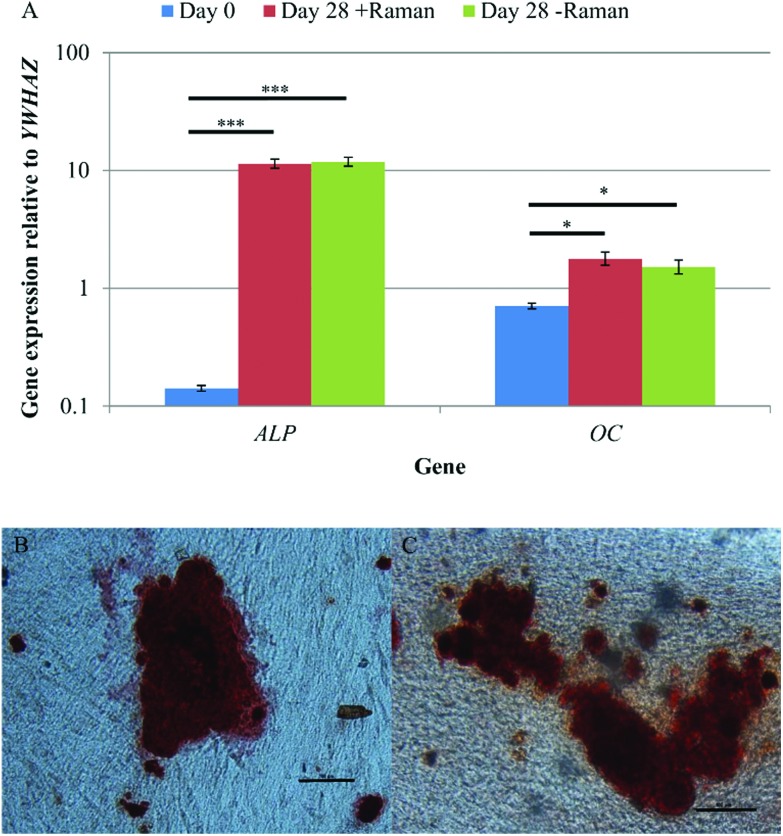
(A) A comparison of DPSC osteogenic gene expression at day 0, and day 28 from cells that had/had not undergone Raman spectroscopy. Results show mean ± SE, *n* = 3 (day 0), *n* = 12 (day 28 + Raman), *n* = 12 (day 28 – Raman). ***p < 0.001, *p < 0.05. Significant increases in the expression of both genes was found between days 0 and 28 and there was no difference in the expression between DPSCs that had and had not undergone Raman spectroscopy. Alizarin red staining of DPSCs after 28 days of osteo-induction that (B) had undergone Raman measurements and (C) had not undergone Raman measurements. Scale bars = 100 μm. Positive staining of mineralised nodules was observed regardless of whether the DPSCs had undergone Raman spectroscopy or not.

qRT-PCR and Alizarin red staining were used to confirm osteoinduction of DPSCs after 28 days in osteoinductive medium. Samples that had undergone Raman measurements, and those grown in parallel without being subject to Raman, were analysed to determine if the data acquisition process and the Raman spectroscopy itself had any effect on DPSC osteogenic differentiation. qRT-PCR showed significant increases in *ALP* and *OC* expression between days 0 and 28 of osteo-inductive culture irrespective of whether or not the DPSCs had undergone Raman analysis (*p* < 0.001 and *p* < 0.05 respectively) (Fig. 7A). There was no significant difference in the expression of either gene between DPSCs which had been analysed by Raman spectroscopy and those that had not.

Alizarin red staining was used to demonstrate extracellular matrix calcification in DPSC cultures after 28 days of osteo-induction for samples that had been analysed by Raman spectroscopy (Fig. 7B) and those that had not (Fig. 7C). Positive Alizarin red staining was observed in all samples, with no discernible differences between the Raman and non-Raman groups. This was not quantified but the findings align with the results of the Raman signature for hydroxyapatite that was obtained from apparent mineralised nodules in the cultures.

### Effect of Raman spectroscopy on DPSC cell stress marker gene expression

**Fig. 8:**
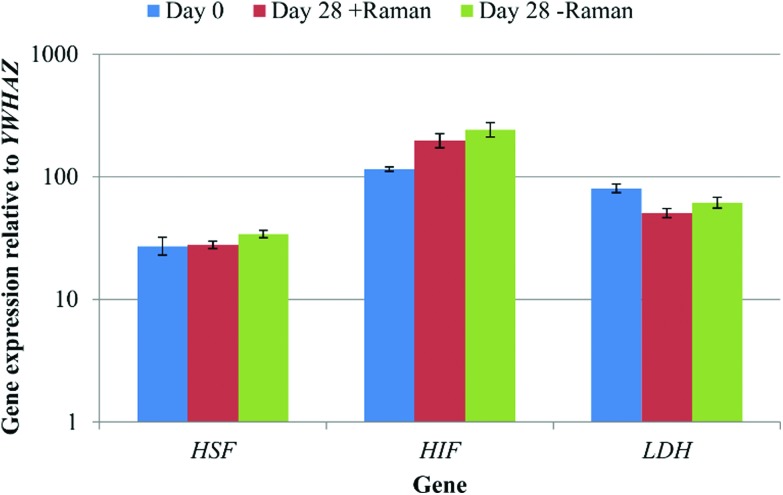
A comparison of DPSC cell stress marker expression at day 0, and day 28 from cells that had/had not undergone Raman spectroscopy. Results show mean ± SE, *n* = 3 (day 0), 12 (day 28 + Raman), 12 (day 28 – Raman). No significant changes in gene expression were observed.

The data acquisition process during Raman spectroscopy used in this study was entirely aseptic and non-invasive permitting the same cell cultures to be analysed repeatedly throughout the differentiation process. However, this method had the potential to induce a cell stress response, due to, for example, limited gas diffusion, exposure to ambient temperature and atmospheric gas concentrations, as well as the laser on the sample. In order to test for any cell stress response, the expression of three marker genes, heat shock transcription factor 1 (*HSF*), hypoxia inducible factor 1 (*HIF*) and lactate dehydrogenase (*LDH*) was determined by qRT-PCR for DPSCs at day 0 and day 28 ± Raman spectroscopy (Fig. 8). No significant differences in the expression of all three genes were observed between the groups (*p* > 0.05) showing that the Raman and the new culturing approach had not affected the cells.

This study had two aims, to develop a method for the aseptic repeated acquisition of Raman spectra from the same cell cultures over a period of time that did not perturb the cells during Raman analysis and to validate this approach by capturing Raman data from DPSCs (a well characterised pro-mineralising stromal cell population) in osteoinductive culture. At the end point of DPSC osteogenic differentiation, mineralised nodules can be clearly seen in the cultures and there is no need for a non-invasive method to determine cellular differentiation status, as such nodules are clearly discernible under an ordinary microscope without staining. Therefore we focused on acquiring spectra in the first 14 days of osteo-induction with a view to identifying spectral features that may be associated with early stage cell differentiation.

The first aim of this study, to acquire Raman spectra repeatedly over time from the same cell cultures under aseptic conditions, was achieved by customising standard tissue culture flasks with quartz windows. This allowed DPSCs to be cultured and analysed in the same vessel making the method truly non-invasive. There was no evidence of any bacterial or fungal contamination of our cultures at any point. However, in future movements towards the clinical application of our research a more extensive and exhaustive demonstration that the technique used is aseptic would be necessary. A lack of optimised environmental control during spectra acquisition had the potential to induce a cell stress response. In order to investigate any cell stress the expression of heat shock transcription factor 1 (*HSF*), hypoxia inducible factor 1 (*HIF1*) and lactate dehydrogenase (*LDH*) was determined by qRT-PCR. *HSF* is a transcription factor regulating the expression of several important heat shock factor proteins including HSP70 and HSP90 ^28^ and whilst it is primarily induced by hyperthermia it has been shown to be activated in response to increasing temperature following hypothermia.^29^ *HIF1* is expressed in response to hypoxia which at physiological levels (2–9% O_2_) is beneficial and a key regulatory mechanism in several biological processes including embryogenesis and stem cell function, however overexpression at pathological levels (<1% O_2_) is associated with several conditions including ischemia and cancer.^30–32^ *LDH* expression is known to be stimulated by *HIF1* expression and is associated with cancer through increased anaerobic glycolysis by a process termed the Warburg effect.^33,34^ No significant differences in the expression of all three marker genes were found regardless of the Raman sampling process and relative to the start of the experiment. Whilst this is not an exhaustive interrogation of the effects of performing Raman spectroscopy on DPSCs in ambient conditions for approximately 3 h at a time, it does indicate that any effects were potentially negligible. Previous studies have also demonstrated that Raman spectroscopy and in particular exposure of cells to near infra-red lasers with a power on the sample of up to 115 mW does not induce cell damage.^35^ Finally, we determined that our method had no effect, detrimental or otherwise, on the osteogenic differentiation process as evidenced by no differences in the expression of osteogenic marker genes or in Alizarin red staining between DPSCs that had or had not undergone analysis by Raman spectroscopy.

The second aim of the study, to validate our aseptic method for cell phenotyping, was achieved using repeated Raman measurements accompanying the osteoinduction of DPSCs over time in culture. PCA was performed on spectra acquired throughout the period in osteoinductive culture. The PCA scores plot of PC1 *versus* PC2 demonstrated two phases in the process hinged at approximately day 10. The loadings for PC1 and PC2 indicated that the pattern of those spectral features indicative of proteins and lipids was similar while features indicative of DNA and RNA had the opposite pattern but were also similar to one another. When the loadings for PC1 and PC2 were plotted against each other, protein associated peaks were shown to vary the most in PC1 and therefore from days 0 to 10 while DNA/RNA associated peaks varied the most in PC2 and therefore from days 10 to 28. It is clear from the averaged spectra at each time point that the protein associated peaks at 1453 cm^–1^ and 1660 cm^–1^ were increasing in intensity. Taken together this suggests that in the early stages of DPSC osteoinduction increasing protein content is the main marker. This is in agreement with the findings of many others^11,13,15^ but is the first instance where this has been demonstrated using repeated measurements taken from the same cell cultures. However, a firm conclusion is more difficult to draw in the case of the DNA/RNA associated peaks contributing to the trend in PC2 due to their relatively low signal intensity in the original spectra and the lower contribution of PC2 (12.4%) to the variation within the data set. Schulze *et al.* 2010 ^11^ found that one of the major indicators of differentiation in embryonic stem cells was the ratio of spectral peaks related to proteins and DNA/RNA, concluding that the intensity of protein associated peaks increased in intensity whilst DNA/RNA peaks decreased in intensity with differentiation. Whilst it is clear from our data that the intensity of protein associated peaks increases with time the trend is less clear with regards to DNA/RNA. Our method however, was clearly able to confirm the presence late stage osteogenic differentiation, when directed toward mineralised nodules as evidenced by the prominent mineral associated region at 955–964 cm^–1^.

## Conclusions

In order to enhance the translation of stem cell based therapies into the clinic, methods that permit the non-invasive characterisation of stem cell cultures prior to their end point are required. Our method, involving the use of standard cell culture flasks customised with quartz windows to permit analysis by Raman spectroscopy is simple, easily reproducible and capable of detecting events that accompany the ultimate osteogenic differentiation of DPSCs. This approach is easily scaled and capable of producing large numbers of Raman-characterised clinically relevant cells. The sampling period in this study was approximately 3 hours per time point/sample to produce 15 spectra for PCA; if the process of acquiring spectra was automated an investigator would only need to prepare the samples. Similarly PCA does not require much time to perform. Taken together the time to perform and analyse Raman spectroscopy would be shorter than to perform and analyse qRT-PCR and staining on an equal number of time points. Using destructive methods such as qRT-PCR on this number of samples and time points would also require a much greater number of cells and materials. As a research tool this method can greatly reduce the time and cost associated with producing and validating osteo-induced stem cells for use in further research such as the production of cell seeded tissue engineered constructs.
